# The Discovery of *Tetracapsuloides bryosalmonae* Infecting Enhanced Migratory Pacific Salmon Populations in the Great Lakes

**DOI:** 10.3390/ani16182948

**Published:** 2026-09-19

**Authors:** Cahya Kurnia Fusianto, Juan-Ting Liu, Bartolomeo Gorgoglione

**Affiliations:** 1 Fish Pathobiology and Immunology Laboratory, Department of Pathobiology and Diagnostic Investigation, College of Veterinary Medicine, Michigan State University, East Lansing, MI 48824, USA; 2 Department of Fisheries and Wildlife, College of Agriculture and Natural Resources, Michigan State University, East Lansing, MI 48824, USA

**Keywords:** Myxozoa, Malacosporea, Chinook Salmon, Coho Salmon, fisheries, fish, reportable pathogen, emerging parasite, P14G8, Proliferative Kidney Disease

## Abstract

Chinook (*Oncorhynchus tshawytscha*) and Coho Salmon (*Oncorhynchus kisutch*) get seasonally restocked in the Great Lakes watershed to support local ecosystems, aquaculture and recreational fishing industry. The myxozoan parasite *Tetracapsuloides bryosalmonae* (Cnidaria, Malacosporea) causes Proliferative Kidney Disease (PKD), a severe chronic lymphoid immunopathology affecting farmed and wild salmonid populations in Central Europe and Northwestern America. We opportunistically sampled migratory Pacific salmon broodstock at fish weirs in Michigan rivers during the autumns of 2020–2023, joining seasonal harvesting campaigns, collecting a total of 591 Chinook and 463 Coho Salmon. *T. bryosalmonae* was detected in broodstock that matured in Lakes Michigan and Huron, with no manifest clinical signs of PKD. Infection prevalence varied among stocks, locations, and years, ranging from 3 to 48% in Coho and 1 to 20% in Chinook. Sequence analysis of *SSU* rDNA and mitochondrial *COI* markers confirmed the detection of *T. bryosalmonae*. Histological examinations revealed both coelozoic sporogonic and histozoic extrasporogonic parasite stages in the posterior kidneys of adult Chinook and Coho. Immunohistochemistry, optimized using a new commercially available *T. bryosalmonae*-specific antibody, further confirmed parasite stages in the anterior and posterior kidney. Further study is needed to characterize this previously undetected, regionally reportable myxozoan parasite and to assess the impact that PKD occurrence may have on fisheries and aquaculture activities in the Great Lakes region. Therefore, tracking this parasite through adequate regulations even in asymptomatic fish hosts is essential, and may guide suitable conservation management actions to safeguard local fish populations and improve preparedness for future potential disease outbreaks.

## Simple Summary

Chinook (*Oncorhynchus tshawytscha*) and Coho (*Oncorhynchus kisutch*) salmon are important fish species in the Great Lakes region. We found them infected with a myxozoan parasite, *Tetracapsuloides bryosalmonae*, upon screening a high number of adult migratory Pacific salmon collected from fish weirs across multiple years and locations in Michigan. This microscopic parasite can cause Proliferative Kidney Disease (PKD), an important disease of salmonids, but the infected adult salmons examined in this study did not show clear signs of disease. Using several laboratory techniques (PCR, histology, immunohistochemistry, and sequence analysis), we confirmed the parasite presence in the kidney of fishes from a large watershed where it has not previously been detected. These findings show that *T. bryosalmonae* can infect Pacific salmon species in the Great Lakes, although its source, distribution, and effects on fish health remain unclear. Environmental conditions in the Great Lakes watershed are changing, creating more favorable conditions for *T. bryosalmonae* transmission, parasite development in freshwater bryozoans, and disease expression in salmonid hosts. Confirming the discovery of the regionally reportable myxozoan parasite *T. bryosalmonae* in fish hosts fills a long-standing gap in the salmonid disease surveillance of the Great Lakes fisheries.

## 1. Introduction

Pacific salmon species, including Chinook (*Oncorhynchus tshawytscha*) and Coho Salmon (*Oncorhynchus kisutch*), were introduced in the Great Lakes in 1966–1967 to control the booming population of highly invasive Alewife (*Alosa pseudoharengus*) [1,2]. They became popular recreational fishery sources in this transboundary region, driving for the development of a large charter industry co-managed by natural resource agencies from all states and provinces that border the Great Lakes, several Native American tribes, and the US Fish and Wildlife Service [3]. During their autumn spawning migration, adult salmon are intercepted at fish weirs installed by the Michigan Department of Natural Resources (MI-DNR) in selected tributaries, where mature broodstocks are collected for hatchery-based stocking programs that help sustain feral populations.

Fish pathogens are a potential key limiting factor for the sustainability of both wild fisheries and regional aquaculture, reducing stock recruitment and triggering strict regulatory measures [2]. Under the Great Lakes Fishery Commission (GLFC) guidelines, reportable fish pathogens are categorized as Emergency, Restricted, or Provisional [4]. Therefore, the Michigan Department of Agriculture and Rural Development (MDARD) lists reportable pathogens and collects data from fish health surveillance of hatchery and feral populations.

*Tetracapsuloides bryosalmonae* is a cnidarian endoparasite (Phylum Myxozoa: Class Malacosporea) that may cause Proliferative Kidney Disease (PKD) in susceptible salmonids [5,6,7]. PKD is considered one of the most severe emerging parasitic freshwater diseases of salmonids in the Northern Hemisphere, causing seasonal mortality in farmed and wild populations in Europe and North America [8,9,10,11,12]. PKD is mainly reported from farmed Rainbow Trout (*O. mykiss*), and is known to have caused wild Brown Trout (*Salmo trutta*) populations to decline in Switzerland [10,13,14]. PKD emergence is strongly dependent on fish susceptibility and on environmental conditions, particularly water temperature [15,16]. Controlled laboratory studies show that constant water temperatures of ≥15 °C strongly increase disease severity and mortality [8,15,17,18], with 30 days at ≥15 °C triggering PKD in Brown Trout [19]. However in cold-water environments, such as large, deep, dimictic lakes, this myxozoan parasite may circulate undetected through subclinical infections and low parasite loads, and it is known to be moving northward [20,21,22]. Even though *T. bryosalmonae* has long been listed by the GLFC among the Emergency Pathogens, and by MDARD among the Regulated Pathogens (to be reported within 24 h), it is not included in any routine surveillance programs in the Great Lakes region [4].

This parasite does not pose a zoonotic risk for fish consumers and cannot be transmitted from fish to fish. The presence of two host types is required to complete its complex life cycle. Indeed, *T. bryosalmonae* exploits freshwater bryozoans as definitive hosts and salmonids as intermediate hosts [5,8,11,23]. In tolerant salmonids, the parasite establishes coelozoic sporogony with minimal impact on host health, producing fish malacospores in the lumen of renal tubules and excreting them through the urinary tract, allowing bryozoan infection to continue their life cycle [5,24,25,26]. In specsies with lower tolerance, it may migrate out of the renal tubules to initiate a histozoic extrasporogonic proliferation in the posterior kidney parenchyma. This aberrant sporulation may trigger PKD, a severe chronic lymphoid immunopathology, eventually resulting in massive renal swelling in highly susceptible fish hosts [15,17,19,27].

In North America, *T. bryosalmonae* was first reported after a PKD-associated mortality event in farmed Rainbow Trout in 1981 in Idaho [28], with subsequent cases reported from California [29], Newfoundland [30], British Columbia [31], Alaska [21], and Montana [9]. In the Summer of 2016, over 6000 Mountain Whitefish (*Prosopium williamsoni*) died in Yellowstone River because of PKD [9], causing the loss of over 500,000 USD following the river closure to recreational activities [32]. Most recently, a severe PKD outbreak led to the closure of a hatchery rearing juvenile Chinook Salmon in Oregon [11]. In the Midwestern region of the USA, this parasite was only reported once, although exclusively detected by PCR from bryozoans from a small hydrologically isolated inland lake in central Michigan (Big Evans Lake) and from bryozoans in a water reservoir connected to the Mad River in Ohio [33]. Nevertheless, no malacosporean infection has ever been reported in fish hosts from this region.

The Laurentian Great Lakes region may currently offer suitable environments and conditions for the completion of the *T. bryosalmonae* life cycle, with both necessary hosts existing, including bryozoans and salmonids (comprising both trout and salmon species), and the water getting seasonally warmer due to climate change trends. However, because this parasite is not included in routine fish health surveillance testing, *T. bryosalmonae* was never reported in any fish host in the Great Lakes watershed.

In this study, we confirm the first compelling evidence of *T. bryosalmonae* infection in adult migratory Pacific salmon in the large Great Lakes region of North America, upon screening specimens collected at MI-DNR fish weirs between 2020 and 2023. This discovery was verified with a solid multi-line-of-evidence approach, using molecular biology techniques, histology, and sequence analysis. Although clinical PKD was not observed in the fish sampled, this was expected as the disease is commonly seen in juveniles [8,14,23,25], which were not available for this study. Our assessment demonstrates that the regionally reportable fish parasite *T. bryosalmonae* is already circulating in the Great Lakes watershed, infecting Pacific salmon species.

## 2. Methods

### 2.1. Fish Sampling, Measurements, and Gross Health Assessment

**Figure 1:**
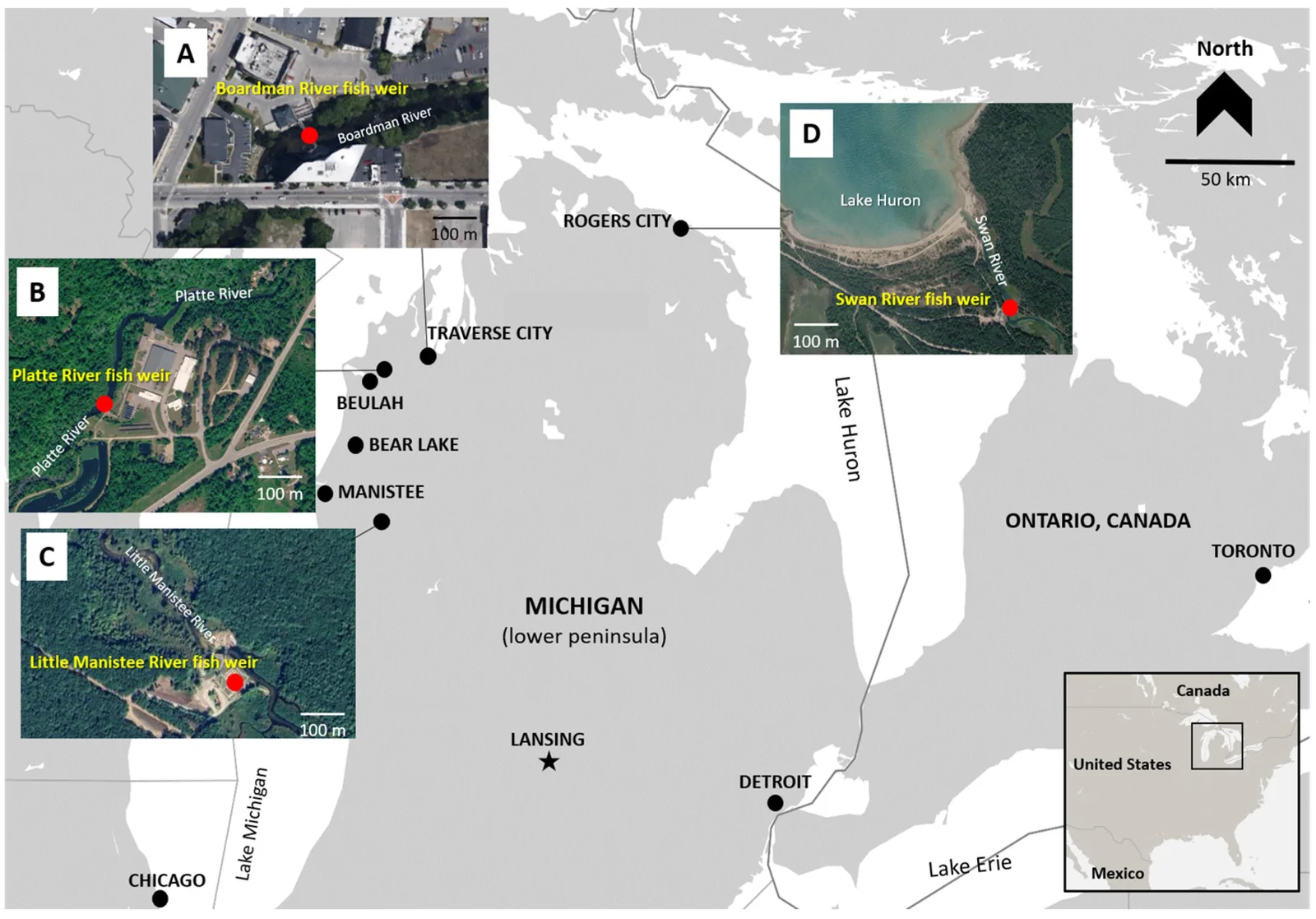
The sampling locations at four MI-DNR fish weirs in Michigan, USA. Red dots indicate each sampling site: Boardman River fish weir (Inset (**A**)), Platte River fish weir (Inset (**B**)), Little Manistee River fish weir (Inset (**C**)), and Swan River fish weir (Inset (**D**)).

Fish were sampled in conjunction with the seasonal fish harvesting and gamete collection operated by the MI-DNR personnel at river weirs across Michigan (Figure 1). Adults and jack broodstocks of two returning Pacific salmon species, including Chinook and Coho Salmon, were sampled during their autumn spawning migrations in October 2020, 2021, 2022, and 2023. Jacks, or precocious “sneaker males”, reach sexual maturity earlier and migrate to spawn one or more years before and at a smaller size than normal. The total length threshold for jacks of Coho Salmon was defined by MI-DNR as <41, <46.6, <53.4, and <48.8 cm, respectively, in 2020, 2021, 2022, and 2023. The total length threshold for jacks of Chinook Salmon was defined as <65.5, <68.4, <73.5, and <65.1 cm, respectively, in 2020, 2021, 2022, and 2023 (MI-DNR, unpublished data). The fish weirs in Boardman River (Traverse City), Little Manistee River (Manistee), and Platte River (Beulahwere built to stop further upstream migration of Pacific salmon from Lake Michigan. The Swan River fish weir stops the upstream salmon migration from Lake Huron, and is located into the world’s largest limestone quarry (Carmeuse Calcite Operation) in Rogers City. At each fish weir, fish organ sampling was carried out in conjunction with the MI-DNR seasonal eggs and sperm collections for the Great Lakes salmon rearing program [1], and the carcasses of all sampled fish were returned to MI-DNR and harvested for industrial processing purposes. From each fish weir location and Pacific salmon species, we aimed to sample 60 fish. In October of 2020 and 2021, due to the repercussions of the global COVID-19 pandemic, sampling was constrained because of limited personnel availability and reduced MI-DNR working capacity. The sample size was calculated to be 95% certain of detecting a pathogen with a minimum expected prevalence of 5% for an unknown population size or more than 100,000 fish [34].

During each field sampling, fish were measured (total body length (TBL), in cm; total body weight (TBW), in grams or Kg) and inspected for any external and internal clinical signs of disease and other morphological alterations, including absence of adipose fin, which is generally used to mark hatchery-sourced salmon. Hatchery-originated Coho could not be distinguished from wild-originated fish because during the COVID-19 pandemic time their adipose fins were not consistently clipped out prior to release.

The gross pathology observation focused on kidney condition, which reflects gross clinical signs of PKD [19,27]. Kidney pathology was graded using the kidney swelling index (KSI), as defined by Clifton-Hadley [35] with modifications [36,37]. Briefly, grade 0 KSI indicates a normal kidney with normal dark red coloration. Grade 1 KSI indicates mild PKD with minimal posterior kidney swelling. Grade 2 KSI indicates progressing PKD with 20–30% kidney swelling. Grade 3 KSI is linked to advanced PKD, with >70% kidney swelling, while grade 4 KSI indicates the most advanced PKD stage, with extensive swelling of the whole kidney with surface corrugations and edematous fluid collections.

### 2.2. Organ Sampling and Preservation

Posterior kidney samples were preserved in RNA-later (Thermo Fisher Scientific, Waltham, MA, USA), kept for 24 h at room temperature, and then stored at −20 °C until processing for molecular biology. Limited to 2021, posterior kidney samples were preserved in 70% ethanol.

Anterior and posterior kidneys, spleen, liver, and gills were preserved in 10% zinc NBF (StatLab, McKinney, TX, USA) for 24 h, thereafter washed under tap water and stored in 70% ethanol until processing for histological examination.

Posterior kidney and spleen organ impression smears were taken from each fish for cytological examination. Each slide was heat-fixed and stored at room temperature before processing, according to the manufacturer’s instructions (Shandon™ Kwik-Diff™ Stains, Thermo Fisher Scientific).

### 2.3. DNA Extraction

A ~20 mg portion (individually weighed) of each posterior kidney specimen was disposed into RNase/DNase-free tubes (2 mL screwcap with O-ring, Fisherbrand, Waltham, MA, USA) with 1.4 mm zirconium ceramic oxide beads (Fisherbrand) and homogenized using a multiple sample homogenizer (Bead Mill 24, Thermo Fisher Scientific) for 1 min at maximum speed (6 m/s). DNA extraction was performed using a Wizard Genomic DNA extraction kit (Promega, Fitchburg, WI, USA), with modifications to the manufacturer’s instructions. Specifically, cell and nuclear lysis buffers were mixed at a 1:3 ratio before tissue homogenization, and the DNA pellet was finally dissolved in sterile TE buffer (pH 8.0). The quality and concentration of each DNA sample was measured spectrophotometrically using an Epoch Microplate Spectrophotometer (Agilent BioTek, Winooski, VT, USA). The DNA samples were stored at −20 °C until use.

### 2.4. Real-Time Quantitative PCR

Real-time quantitative PCR (qPCR) assays were carried out targeting a segment of *T. bryosalmonae* 18s rDNA, according to Gorgoglione et al. [27]. Each sample was run in duplicate qPCR assays, using Quant Studio 3 (Applied Biosystem Waltham, MA, USA). Up to 100 ng DNA was used for each reaction, added with 5 µL of Power Up SYBR Green Master Mix (Applied Biosystem), 0.5 µL of 10 µM of each forward and reverse primer, and PCR-grade water (Fisherbrand) up to a final volume of 10 µL. The qPCR reaction was run with initial denaturation at 95 °C for 10 min, followed by 45 cycles with 95 °C for 15 s and 65 °C for 1 min. The melting curve step was performed at 0.5 °C/s for each subsequent step from 60 °C to 95 °C. Duplicates of the positive control (DNA from the posterior kidney of *T. bryosalmonae*-infected trout from Gorgoglione et al. [38]), negative control (DNA from Common Carp kidney), and no-template control (PCR-grade water) were included in each qPCR assay. For quantification, 7 serial dilutions of the specific purified PCR product, ranging from 1 × 10^6^ to 1 copy, were applied to each qPCR run, as previously defined [38]. Standard dilutions were used to determine the limit of detection (LOD) and limit of quantification (LOQ) according to Sieber et al. [39], resulting respectively in 2 and 5 gene copies per reaction. A positive sample was defined as at least 5 copies of target DNA in one of the two replicates, with the sample melting curve peak within ±1 °C of the melting peak of the positive controls and standard curves.

Ct values were converted to target gene copies/reaction using the standard curve generated in each qPCR run. Copy numbers were calculated from the linear regression equation of the standard curve using QuantStudio™ Design and Analysis software version 1.5.2. Parasite burden was expressed as copies/gram of kidney tissue after adjustment for DNA template volume used in the qPCR, total DNA elution volume, and original mass of kidney tissue extracted.

### 2.5. Conventional PCR

Conventional PCR assays were performed on *T. bryosalmonae* qPCR-positive samples to confirm their positivity and to retrieve parasite-specific Malacosporean small subunit ribosomal DNA (*SSU* rDNA) [40] and mitochondrial cytochrome oxidase subunit 1 (*COI*) [9] sequences. A conventional PCR assay targeting the generic myxosporean 18S rDNA, described by Fiala [41], was also used to screen for the presence of other myxozoan parasites. PCR assays were performed in duplicate using up to 200 ng per reaction, containing 12.5 µL of 2 × JumpStart Red Taq ReadyMix (Sigma-Aldrich, St. Louis, MO, USA), 1 µL each of forward and reverse 10 µM primers (Sigma-Aldrich), and PCR-grade water up to a final volume of 25 µL. Conventional PCR amplifications were performed using a Gene Amp^®^ PCR System 9700 (Applied Biosystem). The specificity of target amplification was ensured in all assays by including suitable positive controls (DNA from the posterior kidney of *T. bryosalmonae*-infected salmon and *Henneguya ictaluri*-infected Catfish for the myxosporean assay), a negative control (DNA from Common Carp kidney and negative salmon for the myxosporean assay), and a non-template control (PCR-grade water). Amplicons were visualized with a UV transilluminator after gel electrophoresis in 1.5% agarose gel at 100 V for 40 min. Further details about each PCR assay, including the specific primers used in this study, are listed in Table 1.

### 2.6. Sequencing and Phylogenetic Analysis

Selected amplicons upon *T. bryosalmonae* positive PCR testing were purified using a QIAquick PCR Purification Kit (Qiagen, Hilden, Germany) and Sanger-sequenced by GENEWIZ-Azenta Life Science, Burlington, MA, USA. The sequencing results were evaluated and trimmed using Bioedit Ver. 7.7 [42]. The similarity of sequences to the reference was assessed using the Basic Local Alignment Search Tool (BLASTn) (https://blast.ncbi.nlm.nih.gov/Blast.cgi). Trimmed nucleotide sequences were aligned using Bioedit and used as an input for phylogenetic tree assembly. A maximum likelihood phylogenetic tree was generated using IQ-TREE [43] with the best-fitting nucleic acid substitution model determined using ModelFinder [44] and 1000 bootstraps by an ultrafast bootstrap approximation approach [45].

### 2.7. Histological Techniques

Histological examination was performed on positive samples selected after qPCR screening. Routine paraffin-embedding and Hematoxylin–Eosin (H&E) staining were performed by the MSU Investigative Histopathology Laboratory, East Lansing, MI-USA using 5 μm sections. Histological specimens were examined using a LABOPHOT-2 microscope (Nikon Corp, Tokyo, Japan) with a SPOT Idea^TM^ CMOS camera (SPOT Imaging, Sterling Heights, MI-USA).

Immunohistochemistry (IHC) was performed in triplicate on selected positive samples in which potential parasitic sporulation was observed in H&E staining. For the presence of *T. bryosalmonae* and confirmation of histological localization, a commercially available polyclonal anti-*T. bryosalmonae* antibody was used. Briefly, 4 μm organ sections were mounted on charged slides and dried overnight at 56 °C. The slides were then deparaffinized in xylene and hydrated using descending amounts of ethyl alcohol up to distilled water. The slides were placed in Tris-buffered saline (TBS) pH 7.4 (ScyTek Laboratories, Logan, UT, USA) for 5 min for pH adjustment. After TBS treatment, the slides were subjected to heat-induced epitope retrieval in a vegetable steamer at pH 6.0, 8.0, and 9.0. After pretreatment, standard avidin–biotin complex staining steps were performed at room temperature using an automated stainer IntelliPATH FLX (Biocare Medical, Pacheco, CA, USA). All staining steps were followed by 2 min rinses in TBS + Tween 20 (ScyTek). Blocking for nonspecific protein was performed with normal goat serum (Vector Labs, Newark, CA, USA) for 30 min, then the sections were incubated for 15 min each with the avidin/biotin blocking system (Avidin D—Vector Labs/d-Biotin, Sigma-Aldrich). The slides were then incubated in primary mouse monoclonal antibody (anti-PKD [P14G8], Vertebrate Antibodies Ltd., Aberdeen, Scotland) diluted 1:100 in normal antibody diluent (NAD) (ScyTek) for 16 h at 4 °C. Biotinylated goat anti-rabbit IgG (H + L) (Vector Labs) was added and incubated for 30 min, followed by addition of avidin–horseradish conjugated reagent (Vector Labs) with 30 min incubation for the visualization of antibody–antigen complexes. For reaction development, a Vector NovaRED^®^ Substrate Kit (Vector Labs), peroxidase chromogen (Vector Labs), and 15 min incubation were used, followed by 15 s counterstaining in Gill’s 1 Hematoxylin (Cancer Diagnostics Inc., Durham, NC, USA) and mounting with synthetic mounting medium. A Sockeye Salmon (*O. nerka*) kidney sample, previously found infected with *T. bryosalmonae* from Alaska [21], was used as a positive control. For negative controls, the samples were incubated with PBS-T instead of with the antibody for each antigen retrieval treatment. All slides were examined and photographed using the microscope indicated above.

### 2.8. Statistical Analysis

Individual parasite loads were analyzed after log-10 transformation to meet the assumption of normality. Parasite burden is presented as a back-transformed geometric mean with a 95% confidence interval. Associations between *T. bryosalmonae* qPCR positivity and fish- or sampling-level variables were assessed using generalized linear models (GLMs). Binomial GLMs with a logit link were fitted separately for each sampling year. No comparisons were made among years, because year-to-year variation may reflect broad temporal, environmental, and sampling-related factors that were not measured in this study. Explanatory variables included Pacific Salmon species, sampling location, sexual dimorphism, fish origin, and male maturity stage, depending on the specific model. Sexual dimorphism was analyzed as male versus female, fish origin as hatchery origin versus wild origin, and male maturity stage as adult males versus jacks.

Fish measurements were used to derive Fulton’s condition factor (K), which is widely used in fisheries and fish biology studies to evaluate the welfare of a fish, calculated using the formula: K = 100 × (W/L^3^), where W is the body weight in grams, and L is the body length in cm [46]. Differences in Fulton’s condition factor between qPCR-positive and qPCR-negative fish were assessed using Gaussian GLMs. This analysis was conducted separately by sampling year, species, and sampling location.

The results from the binomial GLMs are presented as adjusted odds ratios (ORs), 95% confidence intervals (CIs), and *p* values. Odds ratios were calculated by exponentiating model coefficients. The results from the Gaussian GLMs are presented as mean differences in Fulton’s condition factor between qPCR-positive and qPCR-negative fish, with 95% CIs and *p* values. Statistical significance was assessed at α = 0.05. Because these analyses were exploratory and hypothesis-generating, no formal correction for multiple comparisons was applied. Therefore, *p* values should be interpreted as unadjusted, and borderline results near *p* = 0.05 or estimates with wide confidence intervals were interpreted cautiously. All GLMs were fitted using the glm() function in the base R stats package. No additional R packages were used for model fitting or odds-ratio calculation, and output tables were formatted manually. For all binomial GLMs, model convergence, warning messages, unusually large standard errors, and zero-event predictor categories were inspected to assess sparse data and complete or quasi-complete separation. Estimates affected by sparse data or separation were considered not estimable and are reported as NE in the Supplementary Tables S1 and S2. Statistical analyses were performed using R version 4.6.1.

## 3. Results

### 3.1. Samples Retrieval

In the autumn of 2020, organs were collected from a total of 102 Chinook Salmon, including 62 large sexually mature fish and 40 jacks, and 91 originated from hatchery stocks. A total of 46 Coho Salmon were also sampled, including 42 adults and four jacks. Tissue samples from fish captured at the Boardman River and Little Manistee River fish weirs were harvested at a processing facility in Bear Lake, MI (Figure 1).

In the autumn of 2021, due to the difficulties caused by the COVID-19 pandemic, sampling was exclusively carried out at the Platte River fish weir, with 23 adult Coho sampled.

In the autumn of 2022, random samplings were conducted, collecting a total of 204 Coho and 257 Chinook from fish weirs across Michigan. Among the Chinook, 129 were large broodstocks (including 42 of hatchery origin), and 128 were jacks (including 84 of hatchery origin). Among the Coho, 45 were jacks, while 159 were large adults, of which only two were of hatchery origin.

In the autumn of 2023, random samplings were conducted, collecting a total of 194 Coho and 232 Chinook from fish weirs across Michigan. Among the Chinook, 157 were adults, including 70 originating from hatchery, and 75 jacks, including 64 originating from hatchery. Among the Coho, 148 were adults and 46 jacks, including none originating from hatchery. A summary of all fish tissue samples retrieved for this study is provided in Table 2. During some sampling events, jacks were not sampled and are therefore marked as “ns”.

### 3.2. Gross Assessment of PKD-Associated Kidney Swelling

Most sampled fish showed no gross lesions consistent with PKD, although mild kidney swelling was observed sporadically in a small number of fish from each sampling location in 2022 and 2023. In 2022, a grade 1 kidney swelling index (KSI) was recorded in one Chinook jack from the Little Manistee River fish weir and one Coho jack from the Platte River fish weir. Moderate kidney swelling (KSI grade 2–3) was observed in one adult Coho from the Boardman River fish weir, while more severe swelling (KSI grade 3–4) was observed in one Chinook jack from the Swan River fish weir. At the Swan River fish weir, a marked nephrocalcinosis-like lesion was also observed in one hatchery-origin large male Chinook, and Sea Lamprey predation marks were noted in one wild male and one wild female Chinook.

In 2023, mild kidney swelling (KSI grade 1) was observed in adult Chinook from the Boardman, Little Manistee, and Swan River fish weirs, as well as in Chinook jacks from the Little Manistee River fish weir. Grade 1 KSI was also recorded in one large adult Coho from the Platte River fish weir and one Coho jack from the Little Manistee River fish weir. Marked nephrocalcinosis-like kidneys were sporadically observed in hatchery-origin Chinook from the Boardman and Swan River fish weirs.

### 3.3. Tetracapsuloides bryosalmonae Prevalence, Parasite Burden, and Myxosporean Parasite Screening

The qPCR screening revealed the presence of *T. bryosalmonae* DNA in both adults and jacks of Chinook and Coho Salmon at each sampling location in all years when fish were sampled. The overall prevalences of parasite infection in Coho Salmon in 2020, 2021, 2022, and 2023 were respectively 24% (10/42), 48% (11/23), 8% (17/204), and 3% (5/194). Meanwhile in Chinook, overall prevalences were 20% (20/102), 11% (27/257), and 1% (3/232), respectively, in 2020, 2022, and 2023. Details about parasite prevalence at each sampling location and year are shown in Table 3.

In addition, all kidney samples tested negative for the presence of any other Myxozoan parasite, including all specimens resulting as *T. bryosalmonae*-positive, upon the PCR assay targeting generic Myxosporean parasites.

### 3.4. Generalized Linear Model Analysis of Factors Associated with Tetracapsuloides bryosalmonae qPCR Positivity

Year-stratified binomial GLMs evaluated factors associated with *T. bryosalmonae* qPCR positivity within each sampling year. No statistical comparisons were made over the years. Due to limited sample availability in 2021, these samples were excluded from statistical analysis. The full model outputs are provided in Supplementary Table S1.

The fish species was not significantly associated with qPCR positivity in any sampling year. Coho did not differ significantly from Chinook Salmon in 2020 (OR = 0.56, 95% CI: 0.19–1.69, *p* = 0.306), 2022 (OR = 1.58, 95% CI: 0.75–3.33, *p* = 0.229), or 2023 (OR = 1.94, 95% CI: 0.35–10.65, *p* = 0.446). Therefore, within each sampling year, qPCR positivity was not significantly associated with salmon species.

Sampling location was significantly associated with qPCR positivity in 2020 but not in 2022 or 2023. In 2020, fish sampled from the Little Manistee River weir (OR = 0.13, 95% CI: 0.04–0.41, *p* < 0.001) and the Swan River weir (OR = 0.07, 95% CI: 0.01–0.35, *p* = 0.001) had significantly lower adjusted odds of qPCR positivity than fish sampled from the Boardman River weir. The Platte River weir did not differ significantly from the Boardman River weir (OR = 0.47, 95% CI: 0.13–1.72, *p* = 0.256). In 2022, no river weir differed significantly in likelihood of being qPCR-positive for *T. bryosalmonae*. In 2023, no significant location effect was detected, although interpretation was limited due to the low number of qPCR-positive fish and the absence of positives at the Little Manistee River weir. Therefore, evidence for location-associated differences was strongest in 2020, with fish sampled at the Boardman River weir having a higher likelihood of being positive to *T. bryosalmonae*.

Sex-associated differences were evaluated using year-stratified models adjusted for species. Male fish had significantly higher odds of qPCR positivity than female fish in 2022 (OR = 2.64, 95% CI: 1.00–6.92, *p* = 0.049), but not in 2020 (OR = 1.29, 95% CI: 0.39–4.20, *p* = 0.675) or 2023 (OR = 4.85, 95% CI: 0.59–39.85, *p* = 0.142). A sex-associated difference was detected at the unadjusted α = 0.05 threshold only in 2022; however, this result should be interpreted cautiously because the confidence interval was wide and the lower bound was close to 1. Male-only models were used to compare adult males and jacks within each sampling year. Maturity stage was not significantly associated with qPCR positivity in 2020 (OR = 1.99, 95% CI: 0.69–5.74, *p* = 0.205), 2022 (OR = 0.45, 95% CI: 0.20–1.03, *p* = 0.060), or 2023 (OR = 0.63, 95% CI: 0.13–3.02, *p* = 0.567). Therefore, no significant difference in qPCR positivity was detected between adult males and jacks.

Adipose fin clipping was not performed on hatchery-reared Coho Salmon. Therefore, Chinook-only models were used to evaluate the association between fish origin and qPCR positivity to *T. bryosalmonae*. The origin effect could not be estimated reliably in 2020 because no wild-origin Chinook Salmon were qPCR-positive. In 2022, wild-origin Chinook did not differ significantly from hatchery-origin Chinook after adjustment for river weir (OR = 0.79, 95% CI: 0.30–2.06, *p* = 0.630). In 2023, fish origin was also not significantly associated with qPCR positivity (OR = 7.62, 95% CI: 0.54–107.43, *p* = 0.133), although the estimate was imprecise because only three Chinook Salmon were qPCR-positive. Therefore, within the limitations of the available origin data, hatchery or wild origin was not significantly associated with qPCR positivity.

### 3.5. Fulton’s Condition Factor Associated with Tetracapsuloides bryosalmonae qPCR Positivity Analysis

Fulton’s condition factor was analyzed separately by species, sampling year, and river weir to compare qPCR-positive and qPCR-negative fish within the same sampling stratum. Due to limited sample availability in 2021, these samples were excluded from statistical analysis.

The full model outputs are provided in Supplementary Table S2.

In 2020, no significant difference in condition factor was detected between qPCR-positive and qPCR-negative fish in any estimable Chinook or Coho Salmon group. Therefore, qPCR positivity was not associated with detectable differences in body condition in 2020.

In 2022, qPCR-positive Chinook from the Swan River weir had significantly lower condition factors than qPCR-negative Chinook Salmon from the same site (mean difference = −0.123, 95% CI: −0.206 to −0.041, *p* = 0.003). Similarly, qPCR-positive Coho from the Little Manistee River weir had a significantly lower condition factor than qPCR-negative Coho from the same site (mean difference = −0.227, 95% CI: −0.370 to −0.083, *p* = 0.002). No significant condition-factor differences were detected in the remaining estimable species–location groups in 2022. Therefore, lower body condition in qPCR-positive fish was detected in the Swan River and Little Manistee River weirs.

In 2023, qPCR-positive Coho from the Platte River weir had a significantly higher condition factor than qPCR-negative Coho from the same site (mean difference = 0.540, 95% CI: 0.066 to 1.014, *p* = 0.026). However, this result should be interpreted cautiously because only two qPCR-positive Coho Salmon specimens were available in this group. No other significant condition-factor differences were detected in 2023. Therefore, there was no consistent evidence that qPCR-positive fish had lower body condition in 2023. Overall, condition-factor differences were detected only in selected species–year–location strata, and because multiple exploratory comparisons were performed without formal adjustment, these findings should be considered hypothesis-generating rather than definitive evidence of an infection-associated effect on body condition.

### 3.6. Molecular Diagnostic Confirmation and Phylogenetic Analysis

The five qPCR-positive samples with highest estimated parasite burdens from each fish weir were selected for conventional PCR and sequencing. Assays targeted the malacosporean small subunit rDNA (*SSU* rDNA) [40] and mitochondrial cytochrome oxidase 1 (*COI*) [9]. However, *SSU* rDNA and *COI* sequences were successfully obtained only from the highest-burden sample at each location. The remaining subclinical qPCR-positive samples had lower parasite burdens, which likely limited successful amplification and sequencing of these markers.

BLASTN result analysis showed that all our *COI* sequences (554–568 bp) matched 100% with the 53 *T. bryosalmonae COI* reference sequences in GenBank. The 53 reference sequences in GenBank were isolated from Mountain Whitefish (Acc. MT786878-82, MT786885-89, MT786899-900, MT786905, MT786907-13, MT786931-33), Rainbow Trout (Acc. MT786883, MT786891-93, MT786933-35), Brown Trout (Acc. MT786884, MT786881, MT786895-98, MT786922, MT786929-30), Brook Trout (*Salvelinus fontinalis*) (Acc. MT786918-21), and Yellowstone Cutthroat Trout (*O. clarkii bouvieri*) (Acc. MT786906). All the 53 *T. bryosalmonae COI* sequences were sourced from the 2016 PKD outbreak in the Yellowstone River, MT [9].

Our retrieved malacosporean *SSU* rDNA sequence (1668–1683 bp) matched 99.76% to *T. bryosalmonae* reference sequences on GenBank, isolated from Brown Trout (Acc. KF731712) and the bryozoan *Fredericella sultana* (Acc. FJ981823), originating from Scotland and England, respectively (Table 4).

**Figure 2:**
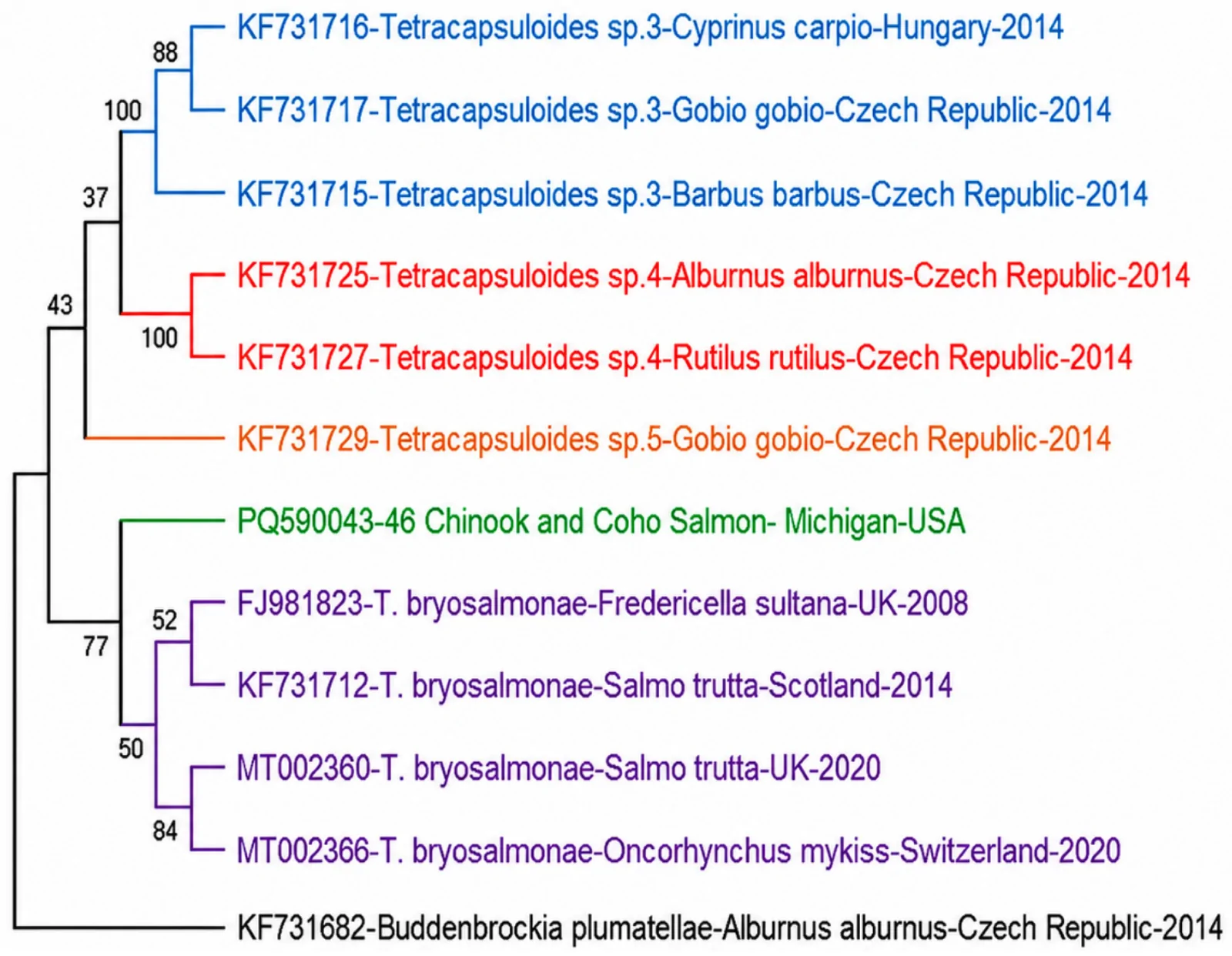
The maximum likelihood cladogram of the partial small subunit (*SSU*) rDNA sequence of *Tetracapsuloides* spp. and the study samples (PQ590043-46 Chinook and Coho Salmon, Michigan). The cladogram included 10 representative *Tetracapsuloides* spp. *SSU* rDNA reference sequences with 1664 nucleotide sites were constructed using a transversion model with empirical frequencies and four gamma distribution categories. The *SSU* rDNA sequence of *Buddenbrockia plumatellae* was used as the outgroup and root for the cladogram. Colors differentiate groups of reference sequences, with samples retrieved from this study shown in green.

**Figure 3:**
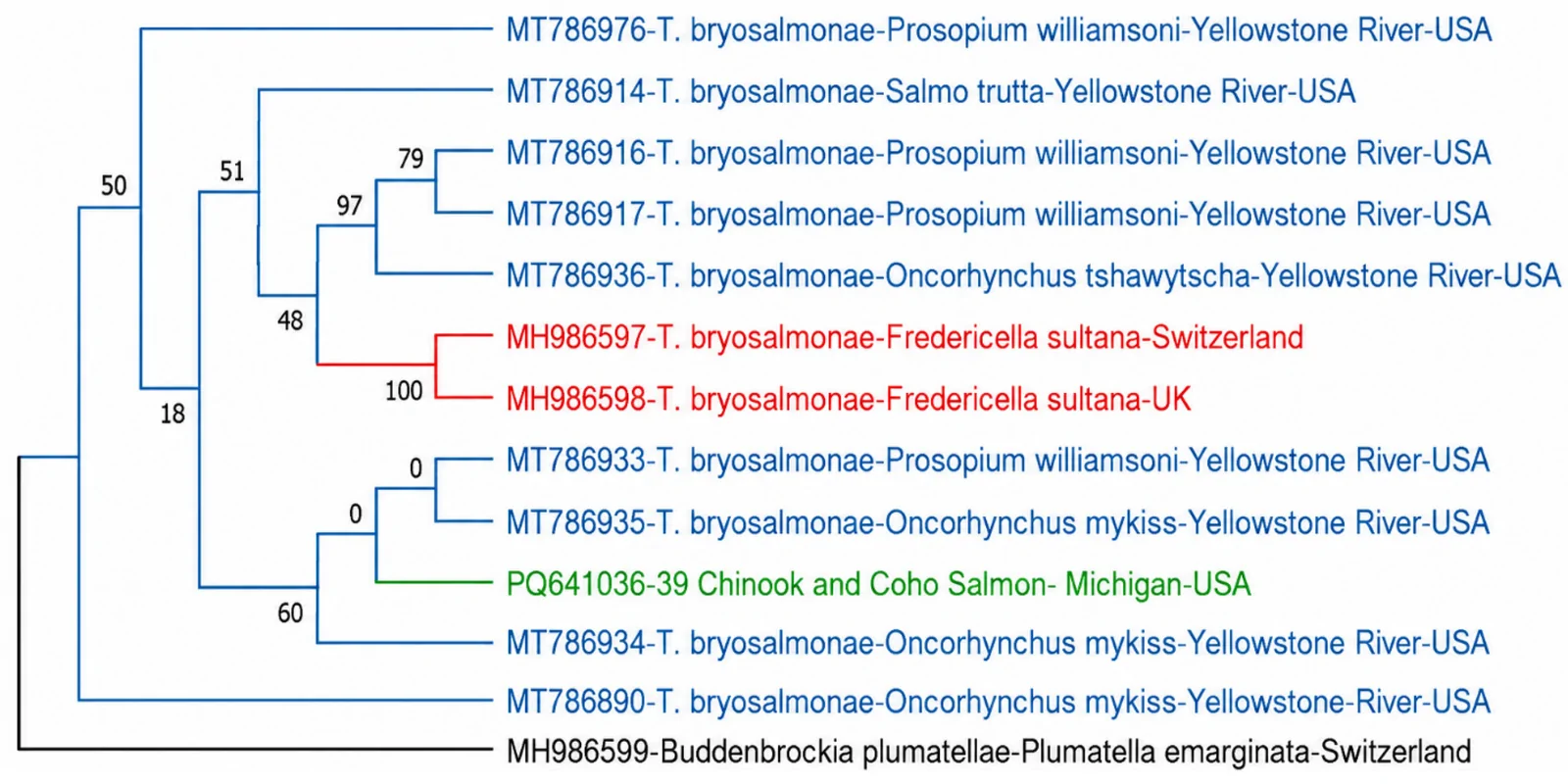
The maximum likelihood cladogram of the mitochondrial cytochrome oxidase 1 (*COI*) sequence of *Tetracapsuloides* spp. and the study samples (PQ641036-39 Chinook and Coho Salmon, Michigan). The cladogram included 10 representative *Tetracapsuloides* spp. *COI* reference sequences with 535 nucleotide sites were constructed using a transition model 2 with unequal base frequencies, empirical frequencies, and an invariable heterogeneity rate. The *COI* sequence of *Buddenbrockia plumatellae* was used as the outgroup and root for the cladogram. Colors differentiate groups of reference sequences, with samples retrieved from this study shown in green.

The cladogram constructed using the *SSU* rDNA showed that our new *T. bryosalmonae* sequences from Michigan were grouped within the group of reference *T. bryosalmonae* strains from Europe but separated from the recently retrieved *Tetracapsuloides* undefined species (sp.3, sp.4, and sp.5). Within the *T. bryosalmonae* reference group, the Michigan sequences also formed a separate cluster from European strains (Figure 2). The malacosporean *COI* cladogram confirmed that our new *T. bryosalmonae* Michigan sequences were identical to some sequences retrieved from the Yellowstone River, MT-USA, but distinct from *T. bryosalmonae* from Switzerland and the UK (Figure 3). However, several deeper nodes in the *SSU* rDNA and *COI* trees had low bootstrap support; therefore, preliminary relationships among Michigan, Yellowstone River, and European lineages should be interpreted cautiously and not over-emphasized.

### 3.7. Cytology Results

No sporoblasts were sensitively identified from impression smears prepared from posterior kidney and spleen specimens. Consequently, this technique proved not to be useful when dealing with carriers or subclinical *T. bryosalmonae* infections and is not further discussed in this study.

### 3.8. Histological Results

Organs collected from fish that tested positive by PCR were further processed for routine histological analysis (using H&E staining), and those with the highest parasite load were further processed for IHC.

**Figure 4:**
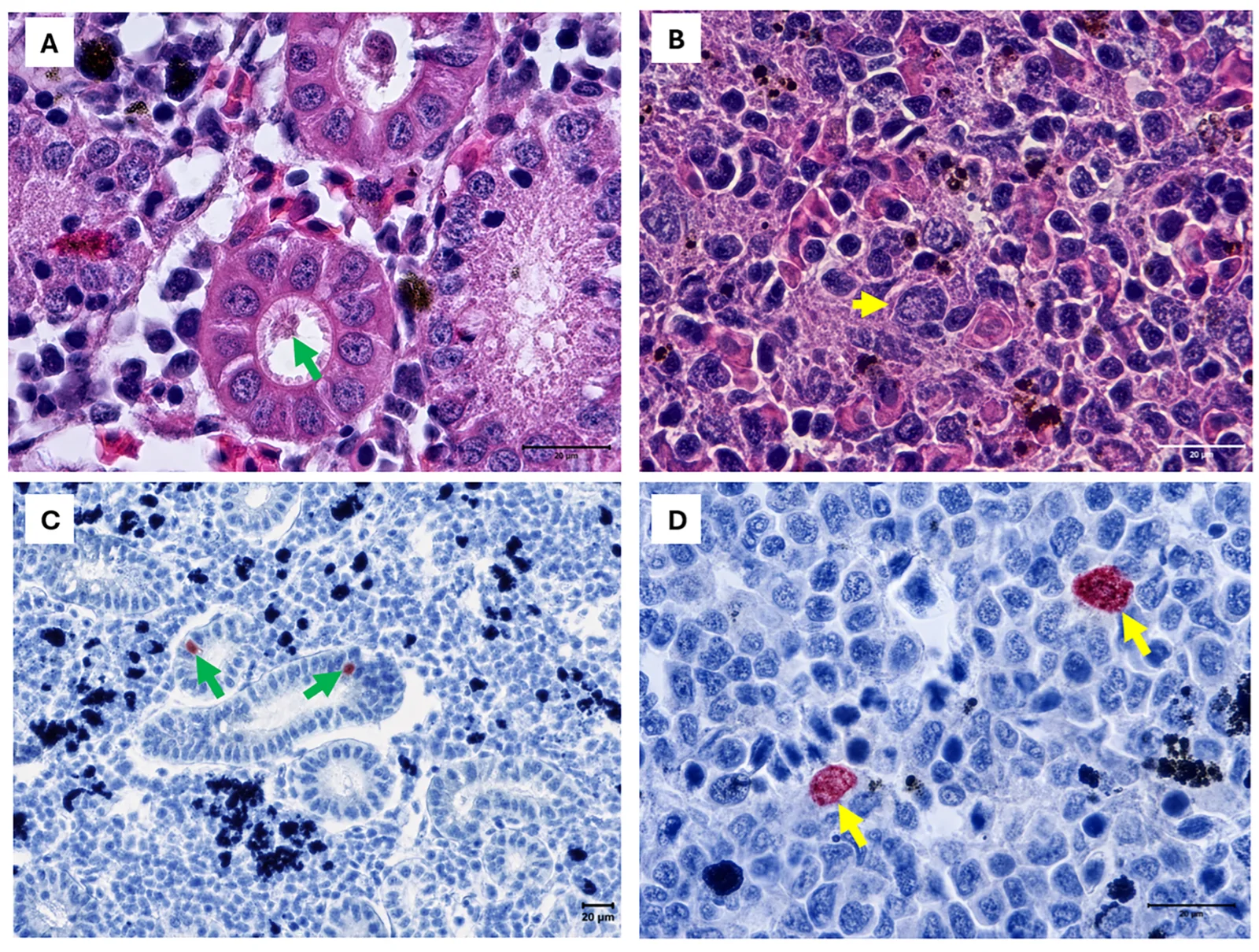
Histological evidence of posterior kidney with coelozoic sporogonic pseudoplasmodia of *Tetracapsuloides bryosalmonae* (green arrows) in the renal tubule lumen of adult Chinook Salmon H&E (**A**) and IHC (**C**), and the histozoic extrasporogonic stage of *T. bryosalmonae* (yellow arrows) in the renal interstitial space, H&E (**B**) and IHC in the anterior kidney (**D**). Bar: 20 µm.

**Figure 5:**
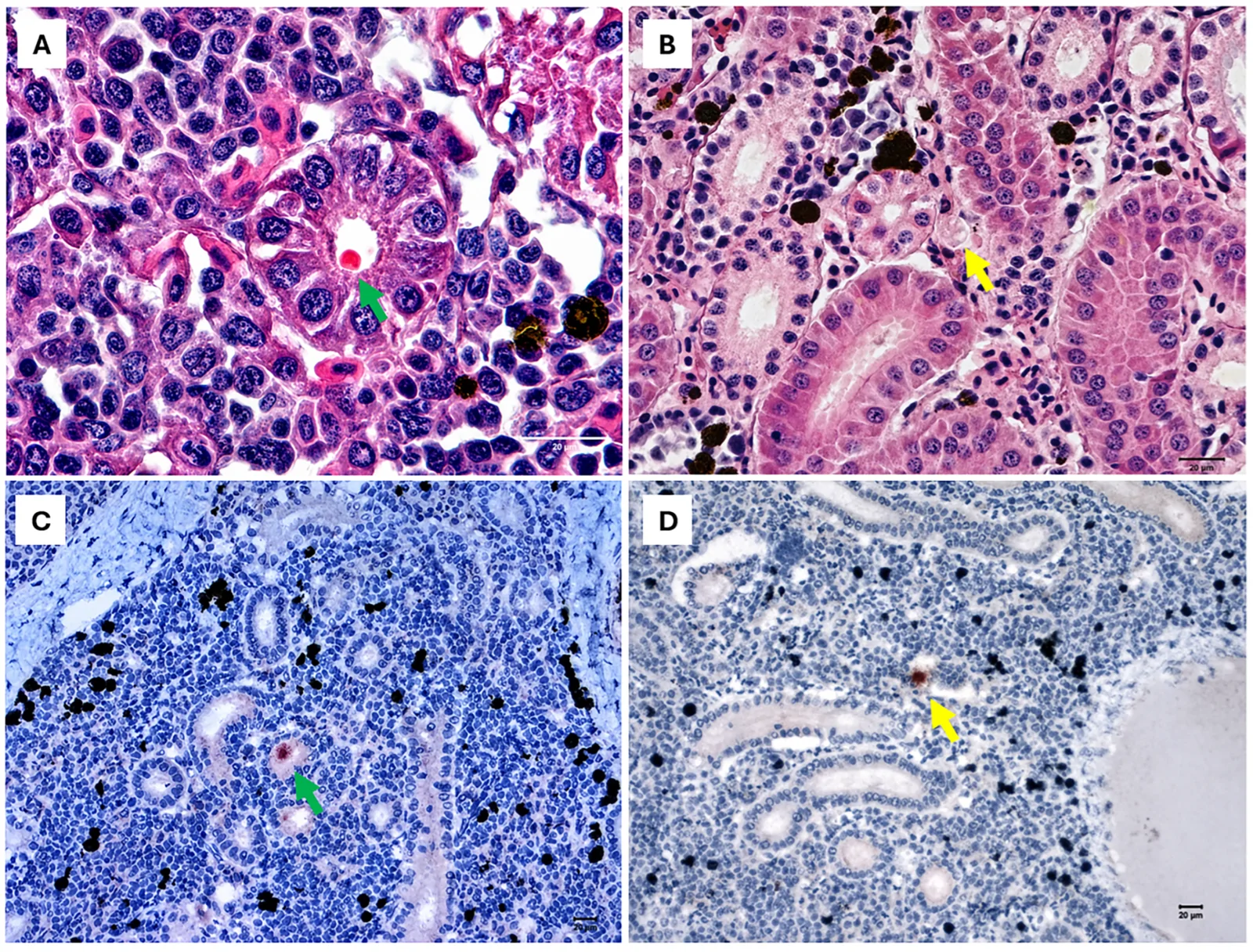
Histological evidence of the posterior kidney with a coelozoic sporogonic stage of *Tetracapsuloides bryosalmonae* (green arrows) in the renal tubule lumen of adult Coho Salmon, H&E (**A**) and IHC (**C**), and a histozoic extrasporonic stage of *T. bryosalmonae* (yellow arrows) in the renal interstitial space, H&E (**B**) and IHC (**D**). Bar: 20 µm.

Routine H&E staining revealed the occurrence of coelozoic sporogonic pseudoplasmodia and histozoic extrasporogonic stages of *T. bryosalmonae* in adults of both Chinook (Figure 4) and Coho Salmon (Figure 5). No host reaction was seen associated with intratubular sporulation, so sporogonic pseudoplasmodia remained localized in the renal lumen of the posterior kidney (Figure 4A and Figure 5A). Extrasporogonic interstitial proliferation was also identified in the renal parenchyma of the posterior kidney, in adults of both Chinook and Coho Salmon (Figure 4B and Figure 5B). In addition, due to a low parasite burden (estimated using qPCR), and the uneven distribution of *T. bryosalmonae* sporoblasts in the kidney, it was difficult to detect any parasite considering that relatively small kidney portions were tested. No histological alterations or specific indications of *T. bryosalmonae* presence were found upon examination of other organs sampled from both fish species, such as in their anterior kidney, spleen, liver, and gills.

IHC was performed on selected higher-load samples confirming the presence of *T. bryosalmonae* in similar locations as observed in corresponding H&E sections. Immunolabeling clearly highlighted sporogonic pseudoplasmodia within the tubular lumen and extrasporogonic stages within the interstitial tissue of the posterior kidney in both adult Chinook (Figure 4C) and Coho Salmon (Figure 5C,D), and in a few cases, within the anterior kidney of adult Chinook (Figure 4D).

## 4. Discussion

This study confirms the first detection of *T. bryosalmonae* infecting adult migratory Chinook and Coho Salmon in the Great Lakes region, detected in specimens from both Lake Michigan and Lake Huron. We identified a subclinical infection in large adult migratory Pacific salmon in both wild and hatchery-reared fish, although PKD was not observed. BLAST analysis of *SSU* rDNA sequences (1784 bp) from the selected positive samples showed 99.76% identity with *T. bryosalmonae*. In addition, BLAST analysis of mitochondrial *COI* gene sequences (619 bp) revealed 100% similarity to 53 *T. bryosalmonae* sequence references in GenBank, although all belonging to *T. bryosalmonae* from the PKD outbreak that happened in the Yellowstone River in 2016 [9].

Detection of *T. bryosalmonae* has been reported mainly in rivers and shallow lakes in Europe and North America [8,10,11,23,47,48,49] and only two studies reported from deep large lakes, including Lake Lucerne in Switzerland in European Whitefish (*Coregonus lavaretus*) [50] and in Brown Trout, Arctic Charr (*Salvelinus alpinus*) and European Whitefish from five deep lakes in Norway [20]. Our study presents the first detection of *T. bryosalmonae* in the Great Lakes region of North America, the largest freshwater reservoir in the world, in adult migratory Chinook and Coho Salmon from deep dimictic lakes, Lake Michigan and Lake Huron. These large dimictic lakes are thermally stratified, with warmer surface water (epilimnion) and colder deep water (hypolimnion) habitats [51,52]. Compared with European studies, where *T. bryosalmonae* infection is commonly associated with Brown and Rainbow Trout in rivers, hatcheries, and smaller lake systems, this study systematically documents subclinical infections in adult migratory Pacific salmon from large, deep, thermally stratified lake systems. Such thermal structure may influence infection outcome because PKD severity is strongly temperature-dependent, whereas cooler temperatures may suppress disease development and favor subclinical infections [15,16,53]. Infected Brown Trout have been shown to prefer colder water [54]. If Chinook and Coho exhibit similar behavior, this could potentially reduce disease insurgence in the Great Lakes region. However, because water temperature and other environmental variables were not part of this study, the role of thermal conditions in modulating *T. bryosalmonae* infection dynamics in Great Lakes salmonids remains unresolved.

Low infection prevalence and parasite burdens near the limit of detection recorded in this study highlight important diagnostic limitations. The focal and uneven distribution of IHC-positive parasite stages suggests that small kidney subsamples may underestimate infection intensity, particularly in subclinical infections with low parasite loads [55]. This limitation is relevant because only a small portion of posterior kidney was used for DNA extraction from large migratory salmon. In addition, the fish sampled at weirs were individuals that had completed migration, whereas severely affected fish may have had reduced swimming performance, increased predation risk, or may have died before reaching the weirs [56]. Because no disease outbreak or mass mortality was observed, infected or dead fish may also have been overlooked in the wild [12,55]. Therefore, infection prevalence in the broader Pacific salmon population in the Great Lakes watershed may be underestimated.

The marked variation in qPCR prevalence among years, species, and sampling locations likely reflects a combination of biological, environmental, and sampling-related factors. Annual differences in water temperature, the timing of salmon migration, bryozoan abundance, and malacospores release may influence exposure risk before fish reach the weirs. Because these environmental and ecological variables were not measured in this study, the observed prevalence patterns should be interpreted as hypothesis-generating rather than evidence of fixed species- or location-specific risk.

Both the sporogonic and extrasporogonic phases of the parasite sporulation activity were observed through histopathology, using routine H&E staining and IHC, highlighting malacospores in the anterior and posterior kidneys of adult Chinook and Coho Salmon. These findings suggest that these Pacific salmon species may act as tolerant or partially susceptible hosts at low water temperatures. Taken together, histological findings with an absence of marked renal enlargement, severe lymphoid hyperplasia, or extensive interstitial inflammation are consistent with a low-intensity, subclinical infection. IHC proved to be particularly useful in samples with low parasite loads, enhancing the visualization of small or isolated parasite stages that were difficult to confidently identify on routine staining alone. Based on the concurrent presence of the sporogonic and extrasporogonic stages, we hypothesize that adult Chinook and Coho Salmon can support the parasite life cycle. However, further work is required to determine whether these fish release viable malacospores capable of infecting bryozoan hosts.

*Tetracapsuloides bryosalmonae* infection has previously been reported in juvenile Chinook and Coho Salmon in North America, including smolts in British Columbia [31] and PKD-associated mortality in hatchery-reared juveniles in California [29]. More recently, PKD was also reported in juvenile Chinook Salmon in Oregon, leading to a temporary hatchery closure [11]. However, to our knowledge, *T. bryosalmonae* infection has not previously been reported in adult/broodstock of either Chinook or Coho Salmon. In this study, *T. bryosalmonae* was detected in adult migratory salmon and jacks, extending current knowledge of host life stages in which infection can be detected. This contrasts with previous North American reports in juvenile Pacific salmon, where *T. bryosalmonae* infection was associated with clinical PKD or mortality in hatchery facilities [11,29,31], suggesting that host life stage, exposure history, and thermal environment may strongly influence disease expression. Although no clinical PKD was observed, differences in Fulton’s condition factor between *T. bryosalmonae*-positive and -negative fish were detected in selected species–location groups. These differences were not consistent across the dataset and should therefore be interpreted cautiously. Overall, the results do not provide strong evidence that infection consistently reduces body condition in adult migratory Chinook or Coho Salmon.

The key factor of *T. bryosalmonae* infection in fish is the presence of their definitive hosts, freshwater bryozoans, as no fish-to-fish transmission happens [5,11,23,57]. The source of *T. bryosalmonae* malacospores infecting Pacific salmon hosts retrieved in this study remains unidentified and will be the object of further environmental studies in Michigan and across the Great Lakes region. Based on our *SSU* rDNA sequence analysis, we hypothesize that infection likely occurs during the early life stages of Pacific salmon in the Great Lakes’ tributary rivers, upon exposure to infectious malacospores released by bryozoan hosts, and can persist through to adulthood. Selected *T. bryosalmonae*-positive Coho and Chinook Salmon collected from each fish weir in 2020 shared comparable *Tetracapsuloides bryosalmonae SSU* rDNA sequences, indicating infection by the same parasite strain. This finding is plausible given that the Coho and Chinook species used for the restocking program are primarily reared in one hatchery supplied with surface water, which may serve as a potential source of infection. Further study on *T. bryosalmonae* infection dynamics in the hatchery is required. In Brown Trout, *T. bryosalmonae* infection can persist for over five years post-exposure, when fish become carriers releasing viable fish malacospores [58]. Another possibility is that the fish host becomes infected by malacospores released by bryozoan hosts in Lakes Michigan and Huron and their tributaries when they are already in the adult stage. Both of these two possibilities are feasible because at least 16 freshwater bryozoan species are known to occur in the Great Lakes and their tributaries, including *Pectinatella magnifica,* two *Fredericella* spp., eight *Plumatella* spp., and *Cristacella mucedo* [59]. Two of them were found PCR-positive for *T. bryosalmonae*, although from local small and isolated water bodies including *Cristatella mucedo* from Big Evans Lake in central Michigan and *Pectinatella* spp. from Cowan and Huffman Lakes in southern Ohio [33]. The detection of *T. bryosalmonae* in Pacific salmon is consistent with exposure within the Great Lakes watershed, but confirmation of an active local life cycle requires direct detection of this parasite in bryozoan hosts and additional salmonid populations. Further study is in progress to determine which bryozoan species act as the main *T. bryosalmonae* hosts in this region. The 100% *COI* similarity between the Michigan and Yellowstone River sequences should not be used to speculate on parasite introduction, mainly because of the very limited *T. bryosalmonae COI* sequence availability in GenBank. Furthermore, the short *COI* fragment sequenced may represent just a partial consensus of a widespread North American haplotype or may have limited marker resolution. More comprehensive multilocus genomic analyses across fish, bryozoan, and environmental samples are needed to assess any likelihood of different introduction pathways such as human-mediated introduction or water flow spread.

Several fish pathogens are known to impact Pacific salmon health in the Great Lakes region, including *Aeromonas salmonicida*, *Renibacterium salmoninarum*, and *Flavobacterium* spp. [60,61]. These bacterial pathogens are included in fish health surveillance programs, which do not include *T. bryosalmonae* despite it has been listed for decades as an Emergency Pathogen by the GLFC, due to its exotic status and potentially severe impact on Great Lakes fish stocks [4]. In this study, we confirm the presence of *T. bryosalmonae* in feral adult migratory Chinook and Coho Salmon, suggesting that its current Emergency Pathogen status needs to be reconsidered. Under the GLFC framework, Provisional Pathogens are pathogens of concern that are not currently classified as Emergency or Restricted, but require further information on their epidemiology, life history, potential effects, and management significance before reclassification can be considered [4]. Therefore, based on the current findings, *T. bryosalmonae* may be more appropriately classified as a Provisional Pathogen at this stage. Thereafter, further epidemiological and pathobiological studies may determine whether to move it among the Level 1 Restricted Pathogens. Level 1 Restricted Pathogens are pathogens demonstrated to be associated with epizootic events or reduced fish fitness in the Great Lakes basin. Fish infected with a Level 1 Pathogen may be stocked in locations where the pathogen is known to be present, and where it may be expected to have minimal impact on infected fish [4]. This new classification should therefore include *T. bryosalmonae* in pre-stocking testing of fish reared in surface water-fed hatcheries. We also suggest MDARD, and corresponding governmental agencies in other states and provinces of the Great Lakes region, moving *T. bryosalmonae* among their Monitored fish pathogens.

In the upper Great Lakes, other salmonid species such as the native Lake Trout (*Salvelinus namaycush*), Cisco (*Coregonus artedi*), and Lake Whitefish (*C. clupeaformis*), as well as introduced species including Rainbow and Brown Trout, Atlantic (*Salmo salar*) and Pink Salmon (*O. gorbuscha*) play important roles in the ecosystem [1,3,59]. Climate projections for the Laurentian Great Lakes indicate upcoming warmer water temperatures, reduced ice cover, longer stratification, and increased thermal stress on cold-water fish habitats [62]. This condition may create more favorable conditions for temperature-dependent waterborne diseases, such as PKD [63]. Further study and targeted pathogen monitoring are required to determine the host status of these salmonid species and any potential negative impacts of this parasite infection to guide suitable management actions in the Great Lakes region. These future studies should integrate environmental monitoring, particularly water temperature, with parasite detection in salmonid hosts, bryozoan populations, and in environmental waters to better evaluate ecological factors and thermal drivers of *T. bryosalmonae* transmission in this area. In addition, questions related to *T. bryosalmonae* strains circulating in the Great Lakes watershed, including pathogenicity and genetic relatedness to the known strains, remain unanswered. This information is essential for disease control and prevention, which is becoming important to conserve fish populations in a changing environment.

## 5. Conclusions

A large-scale survey was carried out, screening a high number of samples across multiple species, years, and locations, searching for the regionally reportable myxozoan parasite *Tetracapsuloides bryosalmonae* in migratory Pacific salmon populations from the Great Lakes. Molecular biology, including qPCR screenings and targeted cPCR followed by sequence analysis, and histological evidence confirmed the occurrence of *T. bryosalmonae* infections in the kidneys of both Coho and Chinook Salmon. This study provides the first confirmed detection of *T. bryosalmonae* in adult migratory Chinook and Coho Salmon broodstock from the large Great Lakes region of North America. Despite the lack of gross clinical signs of PKD found during this study, the occurrence of both sporogonic and extrasporogonic proliferation stages of the parasite was confirmed by IHC, suggesting that mature spores may be released and supporting the occurrence of subclinical infections in adult migratory Pacific salmon. Coho and Chinook from the Great Lakes may act as active hosts, allowing for the propagation of the parasite life cycle. Further studies are needed to determine whether juvenile life stages are susceptible to PKD under permissive environmental conditions. The recategorization of *T. bryosalmonae* from Emergency to Provisional Pathogen and the implementation of the new status, including increasing surveillance of salmonid hosts, bryozoan populations, and environmental water, would help clarify the distribution, persistence, and transmission dynamics of *T. bryosalmonae* in the Great Lakes basin. This data will be important for guiding evidence-based fish health surveillance and management actions.

## Figures and Tables

**Table 1:** Primers and PCR cycling conditions.

Target	Forward Primer (5′–3′)	Reverse Primer (5′–3′)	Amplicon (bp)	Annealing Temperature (°C)	Extension Stage Time (s)	Source
*Tetracapsuloides bryosalmonae* 18S rDNA ^1^	GGACACTGCATGTGCTGCATAGT	CCATGCTAGAATGTCCAGGCACT	215	65	25	[27]
Malacosporean *SSU* rDNA ^2^	CTGCGATGTACTCGTCTTAAAG	CGACCAAGCTCAAACAAGTTT	1784	61	120	[40]
Cytochrome oxidase ^1^ (Nested PCR) ^2^	CATAAGGATATTGGTACTTGGTAT	AGAGATCCAAAAGAACACACCTC	1320	55	90	[9]
1st round						
2nd round	GATATTGCCTGTTTTTGGAC	AGAGATCCAAAAGAACACACCTC	619	55	60	
Generic Myxosporean 18S rDNA ^2^	TTCTGCCCTATCAACTWGTTG	GGTTTCNCDGRGGGMCCAAC	900	57	30	[41]

Note: ^1^ Conventional and qPCR; ^2^ conventional PCR.

**Table 2:** Summary of Coho and Chinook Salmon sampled from Boardman River (**A**), Little Manistee River (**B**), Swan River (**C**), and Platte River (**D**) fish weirs in Michigan during autumn spawning migrations from 2020 to 2023.

Species Sampled	Sampling Date	Total Sampled	Adults								Jacks					
			Sampled	TBL (cm)	TBW (kg)	Condition Factor (K)	Sex		Adipose fin		Sampled	TBL (cm)	TBW (kg)	**Condition Factor (K)**	Adipose Fin	
							Female	Male	Yes	No					Yes	No
(**A**) Boardman River Weir																
Chinook Salmon	10 October 2020	23	15	67.0–101.0	2.88–11.26	1.09 ± 0.23	3	12	0	15	8	41.0–57.0	1.06–4.55	1.01 ± 2.36	0	8
Coho Salmon		15	15	49.0–72.0	1.06–4.55	1.01 ± 0.28	3	12	15	0	0	ns	ns	ns	ns	ns
Chinook Salmon	18 October 2022	77	47	73.0–104.0	5.13–10.87	1.22 ± 0.38	25	22	44	3	30	49.0–67.5	1.05–3.20	1.08 ± 0.11	19	11
Coho Salmon		46	43	52.0–75.0	1.60–5.50	1.14 ± 0.16	21	22	43	0	3	30.0–41.0	0.38–0.79	1.30 ± 0.13	3	0
Chinook Salmon	4 October 2023	75	49	54.0–91.0	2.78–7.84	1.14 ± 0.14	24	25	38	11	26	50.0–65.0	0.92–3.17	1.08 ± 0.16	7	19
Coho Salmon		62	53	48.2–71.1	0.95–3.25	0.92 ± 0.17	35	18	53	0	9	33.1–42.5	0.47–0.76	1.30 ± 0.46	9	0
(**B**) Little Manistee River Weir																
Chinook Salmon	5 and 10 October 2020	46	29	72.5–100.0	1.31–9.21	1.00 ± 0.18	7	22	2	27	17	43.0–57.0	2.58–4.54	1.13 ± 0.16	0	17
Coho Salmon		7	7	59.0–75.0	2.58–4.54	1.11 ± 0.16	2	5	7	0	0	ns	ns	ns	ns	ns
Chinook Salmon	5 October 2022	90	23	53.0–98.0	3.63–10.04	1.09 ± 0.34	7	16	18	5	67	49.0–73.0	1.05–7.26	1.14 ± 0.19	24	43
Coho Salmon		43	40	59.0–78.0	1.73–5.58	1.08 ± 0.17	22	18	38	2	3	41.5–45.0	0.68–0.96	1.02 ± 0.08	3	0
Chinook Salmon	10 October 2023	66	47	67.0–97.0	3.24–9.98	1.01 ± 0.16	31	16	38	9	19	43.0–62.0	0.49–2.31	1.14 ± 0.83	2	17
Coho Salmon		22	18	51.8–64.3	0.96–2.59	0.87 ± 0.14	9	9	18	0	4	39.7–43.5	0.51–0.78	0.83 ± 0.28	4	0
(**C**) Swan River Weir																
Chinook Salmon	14 October 2020	30	15	68.0–99.0	2.95–11.95	0.99 ± 0.11	2	13	4	11	15	48.5–57.0	1.0–1.95	0.95 ± 0.09	2	13
Coho Salmon		1	1	70	2.95	0.86	0	1	1	0	0	ns	ns	ns	ns	ns
Chinook Salmon	12 October 2022	90	59	70.0–102.0	3.5–11.43	1.05 ± 0.12	20	39	26	34	31	45.0–68.0	1.10–3.43	1.11 ± 0.19	1	30
Coho Salmon		16	15	54.0–78.0	1.80–4.26	1.04 ± 0.15	1	15	16	0	1	41.5	0.79	1.11	1	0
Chinook Salmon	11 October 2023	91	61	67.0–99.0	2.90–10.93	1.04 ± 0.22	33	28	11	50	30	48.0–60.2	0.51–2.13	0.83 ± 0.17	2	28
Coho Salmon		20	19	49.0–69.5	0.93–2.79	0.93 ± 0.12	11	8	19	0	1	45	1.09	1.2	1	0
(**D**) Platte River Weir																
Chinook Salmon	5 October 2020	3	3	76.0–84.0	3.92–5.08	0.89 ± 0.12	0	3	3	0	0	ns	ns	ns	ns	ns
Coho Salmon		19	15	43.0–70.5	0.95–3.13	1.056 ± 0.14	5	10	15	0	4	36.0–39.0	0.63–0.77	1.33 ± 0.03	4	0
Coho Salmon	12 October 2021	23	23	55.0–76.0	1.37–3.98	0.93 ± 0.12	11	12	23	0	0	ns	ns	ns	ns	ns
Coho Salmon	19 October 2022	99	61	54.5–78.0	1.41–5.58	0.96 ± 0.14	22	39	61	0	38	34.0–52.0	0.32–1.80	0.95 ± 0.12	38	0
Coho Salmon	24 October 2023	90	58	48.0–72.0	0.51–3.32	0.82 ± 0.18	26	32	58	0	32	22.0–46.4	0.05–0.95	0.79 ± 0.53	32	0

Note: Total body length (TBL) and total body weight (TBW) are shown as a range of minimum and maximum values. Condition factor (K) is shown as mean ± standard deviation. No adipose fin denotes fish of hatchery origin; ns denotes no sample available.

**Table 3:** *Tetracapsuloides bryosalmonae* infection prevalence in Chinook and Coho Salmon collected from multiple fish river weirs across Michigan during their autumn migrations in (**A**) 2020–2021, (**B**) 2022, and (**C**) 2023, calculated upon qPCR screening.

Sampling Year	Weir Location	Chinook Salmon			Coho Salmon		
		Prevalence	Parasite Burden *	95% CI **	Prevalence	Parasite Burden *	95% CI **
(**A**) 2020–2021							
2020	Boardman River	12/23 (52%)	2.44 × 10^5^	6.41 × 10^4^–9.25 × 10^6^	5/15 (33%)	1.21 × 10^5^	7. 59 × 10^4^ –1.93 × 10^5^
	Little Manistee River	6/46 (13%)	1.96 × 10^5^	1.61 × 10^4^ –2.37 × 10^6^	0/7 (0%)	na	na
	Swan River	2/30 (7%)	4.12 × 10^5^	2.59 × 10^5^–6.55 × 10^5^	0/1 (0%)	na	na
	Platte River	0/3 (0%)	na	na	5/19 (26%)	1.52 × 10^5^	6.97 × 10^4^ –3.29 × 10^5^
2021	Platte River	na	na	na	11/23 (48%)	1.51 × 10^6^	2.92 × 10^5^–7.83 × 10^6^
(**B**) 2022							
2022	Boardman River	6/77 (8%)	1.35 × 10^4^	6.03 × 10^3^–3.01 × 10^4^	6/46 (13%)	2.22 × 10^4^	5.62 × 10^3^–8.83 × 10^4^
	Little Manistee River	6/90 (7%)	1.09 × 10^5^	3.12 × 10^4^–3.79 × 10^5^	5/43 (12%)	9.40 × 10^4^	1.98 × 10^4^–4.47 × 10^5^
	Swan River	15/90 (17%)	3.83 × 10^4^	6.67 × 10^3^–2.20 × 10^5^	2/16 (13%)	1.31 × 10^4^	1.02 × 10^4^–1.69 × 10^4^
	Platte River	na	na	na	4/99 (4%)	3.44 × 10^4^	1.66 × 10^4^–7.14 × 10^4^
(**C**) 2023							
2023	Boardman River	1/75 (1%)	8.30 × 10^4^	na	3/62 (5%)	8.19 × 10^4^	2.52 × 10^4^–2.66 × 10^5^
	Little Manistee River	0/66 (0%)	na	na	0/22 (0%)	na	na
	Swan River	2/91 (2%)	3.19 × 10^4^	7.27 × 10^3^–1.40 × 10^5^	0/20 (0%)	na	na
	Platte River	na	na	na	2/90 (2%)	1.99 × 10^5^	4.50 × 10^4^–8.83 × 10^5^

Note: * Parasite burden is indicated as geometric mean/g of kidney tissue, with ** 95% confidence interval; na denotes not available data, e.g., because no positive was found or because no Chinook Salmon was retrieved from Platte River fish weir from 2021 to 2023.

**Table 4:** The results of the Basic Local Alignment Search Tool (BLAST) of the *Tetracapsuloides bryosalmonae* small subunit rDNA and mitochondrial cytochrome oxidase I gene sequence from positive samples retrieved in this study.

Weir Location	Host Species	Sequencing of *SSU* rDNA				Sequencing of *COI*			
		Acc Number (From This Study)	Closest Published *SSU* rDNA Sequence			Acc Number (From This Study)	Closest Published *COI* Sequence		
			Organism	Identity ^1^	Acc Number (NCBI References)		Organism	Identity ^2^	Acc Number
									(NCBI References)
Boardman River	Chinook Salmon	PQ590043	*T. bryosalmonae*	99.76%	KF731712.1 and FJ981823.1	PQ641036	*T. bryosalmonae*	100%	MT786878.1–MT786935.1
Little Manistee River	Chinook Salmon	PQ590045	*T. bryosalmonae*	99.76%		PQ641038	*T. bryosalmonae*	100%	
Swan River	Chinook Salmon	PQ590044	*T. bryosalmonae*	99.76%		PQ641039	*T. bryosalmonae*	100%	
Platte River	Coho Salmon	PQ590046	*T. bryosalmonae*	99.76%		PQ641037	*T. bryosalmonae*	100%	

Note: ^1^ Percent identity derived from 1668 bp sequence at the nucleotide level of a sample selected from each sampling location. ^2^ Percent identity derived from 554 bp sequence at the nucleotide level of a sample selected from each sampling location.

## Data Availability

The data that support the findings of this study are available in the Supplementary Materials of this article.

## Supplementary Materials

The following supporting information can be downloaded at https://www.mdpi.com/article/10.3390/ani16182948/s1, Supplementary Table S1: Year-stratified binomial generalized linear model analysis of factors associated with *Tetracapsuloides bryosalmonae* qPCR positivity in migratory Pacific salmon sampled at Michigan river weirs. Supplementary Table S2: Species-, year-, and location-stratified Gaussian generalized linear model analysis comparing Fulton’s condition (K) factor between *T. bryosalmonae* qPCR-positive and qPCR-negative Pacific salmon.
