# Supplementary material for: The Discovery of Tetracapsuloides bryosalmonae Infecting Enhanced Migratory Pacific Salmon Populations in the Great Lakes
Source: Animals (Basel). 2026 Sep 19;16(18):2948. doi: 10.3390/ani16182948 (PMC13603474; doi:10.3390/ani16182948)
Supplement: Supplementary file 1 [file animals-16-02948-s001.zip › animals-4492870-supplementary.pdf]

Supplementary Table S1. Year-stratified binomial generalized linear model analysis of factors associated with *Tetracapsuloides bryosalmonae* qPCR positivity in migratory Pacific salmon sampled at Michigan river weirs.

Sampling year	Predictor	Comparison	Compared Group qPCR+/n (%)	Reference Group qPCR+/n (%)	Adjusted OR (95% CI)	*p*-value
2020	Species	Coho vs Chinook	10/42 (23.8%)	20/102 (19.6%)	0.56 (0.19–1.69)	0.306
	River weir	Little Manistee vs Boardman	6/53 (11.3%)	17/38 (44.7%)	0.13 (0.04–0.41)	**<0.001**
	River weir	Swan vs Boardman	2/31 (6.5%)	17/38 (44.7%)	0.07 (0.01–0.35)	**0.001**
	River weir	Platte vs Boardman	5/22 (22.7%)	17/38 (44.7%)	0.47 (0.13–1.72)	0.256
	Sex	Male vs female	26/122 (21.3%)	4/22 (18.2%)	1.29 (0.39–4.20)	0.675
	Maturity stage	Jack vs adult male	10/44 (22.7%)	16/78 (20.5%)	1.99 (0.69–5.74)	0.205
	Origin	Wild vs hatchery	0/11 (0.0%)	20/91 (22.0%)	NE	NE
2022	Species	Coho vs Chinook	17/204 (8.3%)	24/257 (9.3%)	1.58 (0.75–3.33)	0.229
	River weir	Little Manistee vs Boardman	10/133 (7.5%)	12/123 (9.8%)	0.77 (0.32–1.86)	0.56
	River weir	Swan vs Boardman	15/106 (14.2%)	12/123 (9.8%)	1.71 (0.74–3.93)	0.21
	River weir	Platte vs Boardman	4/99 (4.0%)	12/123 (9.8%)	0.30 (0.09–1.02)	0.054
	Sex	Male vs female	36/343 (10.5%)	5/118 (4.2%)	2.64 (1.00–6.92)	**0.049**
	Maturity stage	Jack vs adult male	11/171 (6.4%)	25/171 (14.6%)	0.45 (0.20–1.03)	0.06
	Origin	Wild vs hatchery	10/131 (7.6%)	14/126 (11.1%)	0.79 (0.30–2.06)	0.63
2023	Species	Coho vs Chinook	5/192 (2.6%)	3/232 (1.3%)	1.94 (0.35–10.65)	0.446
	River weir	Little Manistee vs Boardman	0/88 (0.0%)	4/136 (2.9%)	NE	NE
	River weir	Platte vs Boardman	2/90 (2.2%)	4/136 (2.9%)	0.55 (0.09–3.48)	0.523
	River weir	Swan vs Boardman	2/110 (1.8%)	4/136 (2.9%)	0.75 (0.12–4.53)	0.75
	Sex	Male vs female	7/256 (2.7%)	1/168 (0.6%)	4.85 (0.59–39.85)	0.142
	Maturity stage	Jack vs adult male	3/143 (2.1%)	4/113 (3.5%)	0.63 (0.13–3.02)	0.567
	Origin	Wild vs hatchery	2/98 (2.0%)	1/134 (0.7%)	7.62 (0.54–107.43)	0.133

Note: OR, odds ratio; CI, confidence interval; NE, not estimable due to sparse data or complete separation. Binomial generalized linear models with logit link were fitted separately for each sampling year; no statistical comparisons were made among years. Chinook Salmon, Boardman River weir, female fish, adult males, and hatchery-origin Chinook Salmon were used as reference categories where applicable. The qPCR+/n columns shows the number of qPCR-positive fish divided by total number of fish in each compared or reference group, with raw prevalence in parentheses. Significant *p* values are shown in bold.

Supplementary Table S2. Species-, year-, and location-stratified Gaussian generalized linear model analysis comparing Fulton's condition (K) factor between *Tetracapsuloides bryosalmonae* qPCR-positive and qPCR-negative Pacific Salmon.

Sampling Year	Species	River Weir	qPCR-positive (n)	qPCR-negative (n)	Mean K Positive	Mean K Negative	Mean Difference (95% CI)	*p*-value
2020	Chinook	Boardman	12	11	1.139	1.088	0.051 (-0.118 to 0.220)	0.553
	Chinook	Little Manistee	6	40	1.086	1.042	0.044 (-0.111 to 0.199)	0.578
	Chinook	Swan	2	28	1.002	0.971	0.030 (-0.113 to 0.174)	0.681
	Coho	Boardman	5	10	0.997	1.021	-0.024 (-0.333 to 0.286)	0.881
	Coho	Platte	5	14	1.213	1.08	0.133 (-0.034 to 0.300)	0.118
2022	Chinook	Boardman	6	71	1.047	1.177	-0.130 (-0.391 to 0.131)	0.328
	Chinook	Little Manistee	5	85	1.042	1.134	-0.092 (-0.308 to 0.124)	0.406
	Chinook	Swan	13	77	0.963	1.086	-0.123 (-0.206 to -0.041)	**0.003**
	Coho	Boardman	6	40	1.191	1.148	0.044 (-0.095 to 0.183)	0.539
	Coho	Little Manistee	5	38	0.874	1.101	-0.227 (-0.370 to -0.083)	**0.002**
	Coho	Platte	4	95	0.968	0.966	0.002 (-0.131 to 0.135)	0.975
	Coho	Swan	2	14	0.995	1.048	-0.052 (-0.280 to 0.176)	0.654
2023	Chinook	Swan	2	89	1	0.973	0.027 (-0.293 to 0.347)	0.868
	Coho	Boardman	2	42	0.927	1	-0.073 (-0.485 to 0.338)	0.727
	Coho	Platte	2	88	1.338	0.798	0.540 (0.066 to 1.014)	**0.026**

Note: K, Fulton's condition factor; CI, confidence interval. Gaussian generalized linear models with identity link were fitted separately within each species, sampling year, and river weir. The model compared Fulton's condition factor between qPCR-positive and qPCR-negative fish within the same sampling stratum. The mean difference represents mean K in qPCR-positive fish minus mean K in qPCR-negative fish; negative values indicate lower mean condition factor in qPCR-positive fish. No statistical comparisons were made among years, species, or river weirs. Groups with no qPCR-positive fish, no qPCR-negative fish, or insufficient data for model estimation are not shown. Significant *p* values are shown in bold.
